# New-Onset Diabetes as a Red Flag for Pancreatic Cancer: From Metabolic Clues to Earlier Detection in Primary Care

**DOI:** 10.7759/cureus.117696

**Published:** 2026-10-09

**Authors:** Ludwig Camacho-Flores, Alejandro Alvarez, Rafael Bonet Vazquez, Patricia Benet, Carmen Bárbara Alfonso García, Merlin Marti del Rio

**Affiliations:** 1 General Medicine, University of the Andes, Merida, VEN; 2 Internal Medicine, Ross University School of Medicine, Bridgetown, BRB; 3 Internal Medicine, Universidad de Ciencias Médicas de Las Tunas, Las Tunas, CUB; 4 General Practice, Universidad Miguel Hernández de Elche, Alicante, ESP; 5 Internal Medicine, Universidad de Ciencias Médicas de Granma, Manzanillo, CUB

**Keywords:** biomarkers, early detection, endpac, new-onset diabetes, pancreatic cancer, pancreatic ductal adenocarcinoma, primary care, risk stratification

## Abstract

Pancreatic ductal adenocarcinoma (PDAC) is usually diagnosed at an advanced stage and carries a poor prognosis. In a subset of patients, new-onset diabetes may be an early metabolic manifestation preceding the cancer diagnosis, and its recognition in primary care could help identify people at increased risk, although the benefit of using this association for earlier detection remains uncertain. This narrative review examines the clinical and biological relationship between new-onset diabetes and PDAC and evaluates current strategies for risk stratification, including clinical risk scores such as the Enriching New-onset Diabetes for Pancreatic Cancer (ENDPAC) score, circulating biomarkers, polygenic scoring, and machine-learning models, to identify high-risk populations that could benefit from early detection and to assess whether these tools are ready for routine use. PDAC-associated diabetes differs from typical type 2 diabetes: it tends to occur in older individuals, is accompanied by unexplained weight loss, and progresses more rapidly. Risk-prediction scores perform well in their derivation cohorts but discriminate less well on external validation, and candidate biomarkers, alone or in multi-marker panels, show promising but not yet prospectively confirmed discrimination. Models integrating clinical and genetic data also show promising discrimination, yet none has been shown to detect PDAC earlier or to improve survival. New-onset diabetes may therefore be a warning sign of pancreatic cancer, but current evidence does not support screening of all affected patients; clinical features, biomarkers, and genetic information may help identify a higher-risk subgroup, and further studies are needed before these tools enter routine practice.

## Introduction and background

In the United States, pancreatic cancer is the third leading cause of cancer mortality, with an estimated 67,530 new cases and 52,740 deaths expected in 2026 [1]. Its outlook differs sharply from most cancers: although five-year relative survival for all cancers combined reached 70% for cases diagnosed between 2015 and 2021, the rate for PDAC remains about 13% [1]. Globally, an estimated 510,529 new cases and 467,290 deaths occurred among adults aged 20 years or older in 2022; the mortality-to-incidence ratio approached one in most regions, and projections suggest rising case and death counts through 2045 [2].

Prognosis depends heavily on stage at diagnosis. Of cases diagnosed between 2018 and 2022, only 17% were localized, and 46% had distant metastasis, with five-year relative survival of 44% and 3%, respectively [1]. Specific symptoms tend to appear late: in retrospective reviews of computed tomography (CT) performed before diagnosis, the tumor was undetected or still resectable on scans obtained more than six months before clinical diagnosis, and symptoms often coincided with radiological signs of unresectability [3]. Population-wide screening is not feasible because the low prevalence of the disease would generate unacceptable numbers of false positives [4]. In 2019, the US Preventive Services Task Force reaffirmed its Grade D recommendation against screening asymptomatic adults, including those with new-onset diabetes, excluding only certain hereditary or familial high-risk groups [5]. Identifying subgroups at sufficiently high risk to warrant targeted evaluation is therefore a prerequisite for earlier detection. Importantly, this recommendation addresses untargeted screening of the asymptomatic general population and does not speak to targeted enrichment, the fundamentally different strategy of concentrating evaluation on a biomarker- or phenotype-defined high-risk subgroup, which is the focus of this review.

New-onset hyperglycemia offers one route to such subgroups. A case-control study found that 47% of patients with pancreatic cancer were diabetic compared with 7% of controls, and among those with known diabetes duration, roughly 75% had less than two years of diabetes [6]. The relationship is temporal: mean fasting glucose did not differ between cases and controls more than 36 months before diagnosis, differences emerged approximately 30 to 36 months before diagnosis, and the group means crossed the diabetic threshold 6 to 12 months before diagnosis [7]. Consistent with this, while type 2 diabetes of more than five years' duration carries a risk 1 to 1.5 times that of the general population, risk rises to about 5.4-fold in those with less than one year of diabetes [4]. Prospective data agree: in an interim analysis of 18,838 adults aged 50 years or older with glycemically defined new-onset diabetes, the adjusted three-year incidence of pancreatic cancer was 0.62%, the standardized incidence ratio was 4.6, and the median interval between glycemic onset and cancer diagnosis was 8.1 months [8]. Long-standing diabetes is thus associated with a modestly increased risk, whereas new-onset diabetes may be a manifestation of the tumor in a subset of patients. Unlike broader syntheses of pancreatic cancer biology, this review is framed around the primary care encounter, where adult-onset hyperglycemia is first detected and most often managed as ordinary type 2 diabetes without suspicion of occult malignancy. The following sections examine the mechanisms of this association, the clinical features that may distinguish cancer-associated new-onset diabetes from type 2 diabetes, the realistic barriers to imaging-based evaluation, contemporary risk-prediction models, and the implications for earlier detection in primary care.

## Review

Methodology

This structured narrative review followed the Scale for the Assessment of Narrative Review Articles (SANRA) rather than a systematic review. We searched PubMed/MEDLINE from database inception through 2026 for English-language articles on the relationship between new-onset diabetes and pancreatic cancer, combining terms for new-onset diabetes, pancreatogenic (type 3c) diabetes, pancreatic ductal adenocarcinoma, risk stratification and prediction models (including Enriching New-onset Diabetes for Pancreatic Cancer (ENDPAC)), biomarkers, genetics, machine learning, and early detection; the reference lists of retrieved articles and relevant reviews were hand-searched to mitigate reliance on a single database. Eligible records were epidemiological reports, cohort and case-control studies, systematic reviews and meta-analyses, biomarker and prediction model studies, mechanistic investigations, and clinical guidelines that addressed the association between new-onset diabetes and pancreatic cancer, its biology, clinical recognition, risk stratification, or early detection. Priority was given to contemporary evidence, with seminal earlier work retained where it established a concept still in use. We excluded records addressing diabetes treatment or the general management of pancreatic cancer without relevance to this association. Two authors screened records by title and abstract and assessed full texts against these criteria, resolving disagreements through discussion. Consistent with a narrative design, we did not apply a formal risk-of-bias instrument, certainty-of-evidence grading, a Preferred Reporting Items for Systematic Reviews and Meta-Analyses (PRISMA) flow diagram, or quantitative synthesis; we weighed study type and methodological transparency qualitatively when appraising each source, and we acknowledge this as a limitation.

Biological link: why does pancreatic cancer cause new-onset diabetes?

Substantial evidence supports the view that the diabetes accompanying PDAC is a paraneoplastic, type 3c (pancreatogenic) phenomenon rather than coincidental type 2 diabetes [9]. Hyperglycemia in PDAC arises from a combination of progressive destruction of functional pancreatic tissue and pronounced peripheral insulin resistance [10]. Tumor invasion, extensive desmoplastic remodeling, and ductal obstruction reduce microcirculatory flow and increase islet hypoxia [10], and even before clinical detection, patients with PDAC show marked insulin resistance in the liver and skeletal muscle [11]. Peripheral resistance drives compensatory hyperinsulinemia, but continued beta-cell loss reduces functional beta-cell mass, making insulin secretion insufficient and leading to diabetes [11].

Tumor-host interactions disrupt glucose regulation globally [12]. The metabolic drain imposed by the tumor affects the whole-body energy economy [13]: tumor cells foster a microenvironment that suppresses hepatic gluconeogenesis while increasing catabolism in other tissues, creating a hypermetabolic state that impairs glucose regulation [13]. Beyond effects on hepatic gluconeogenesis and tissue breakdown, PDAC also perturbs the entero-insular axis, impairing incretin signaling through glucagon-like peptide-1 and glucose-dependent insulinotropic polypeptide, while elevated circulating amino acids and lipids further compromise glucose-dependent insulin secretion [14].

At the molecular and cellular level, pro-inflammatory signaling and tumor-secreted factors drive this paraneoplastic progression [15]. PDAC cells overproduce adrenomedullin, a regulatory peptide that both inhibits insulin secretion by acting directly on beta cells and increases insulin resistance in skeletal muscle [16]. Tumor-derived extracellular vesicles deliver bioactive cargo, including proteins such as S100A9 and microRNAs, which, when taken up by islet beta cells, induce endoplasmic reticulum stress, apoptosis, and reduced insulin production [15]. Cancer-associated systemic inflammation, with elevated tumor necrosis factor-alpha and interleukin-6, interferes with post-receptor insulin signaling through serine/threonine phosphorylation of insulin receptor substrate-1 [9].

Clinical phenotypes of new-onset diabetes associated with pancreatic cancer

Recognizing the clinical features of PDAC-associated new-onset diabetes could reduce late diagnosis, and distinguishing it from classic type 2 diabetes, together with identifying risk factors, helps clarify which patients warrant concern [17]. The pathophysiology differs: diabetes is present in a substantial proportion of patients with pancreatic cancer, and the subset that is paraneoplastic and new-onset can resolve after tumor resection; this PDAC-associated new-onset diabetes, rather than diabetes accompanying PDAC in general, is characterized chiefly by beta-cell dysfunction with insufficient insulin secretion, with insulin resistance present but less pronounced than in type 2 diabetes [18]. Although hyperglycemia is common to both, the context differs: patients with PDAC-associated new-onset diabetes tend to be older and to show metabolic features atypical of type 2 diabetes, and weight loss and rapidly rising glycemia are important clues, so the clinical phenotype and accompanying changes in weight and glycemic control may identify a higher-risk subgroup [19,20].

Risk is highest within the first two to three years after diabetes diagnosis [21,22]. New-onset diabetes accompanied by weight loss, older age, a previously healthy and stable weight, and a family history of diabetes has been associated with a substantially increased risk of pancreatic cancer [22,23], and new-onset diabetes in PDAC is typically preceded by a rapid rise in glucose despite weight loss [8]. New-onset diabetes diagnosed after age 50 has served as an early indicator relative to general population risk [24]. Particular attention is warranted for atypical metabolic presentations, such as unintentional weight loss with a rapid rise in glucose and glycated hemoglobin (HbA1c), and a personal history of pancreatitis or gallbladder disease or a family history of pancreatic cancer may further increase the likelihood; recognizing this high-risk phenotype offers a rational first step toward targeted evaluation in primary care [19]. In summary, older age at onset, unintentional weight loss in a previously weight-stable person, and worsening glycemic control raise suspicion, and although these features are not diagnostic, they help separate patients with a high probability of underlying malignancy from those with conventional type 2 diabetes (Table 1).

**Table 1:** Clinical features distinguishing pancreatic cancer-associated new-onset diabetes from typical type 2 diabetes. These features are not individually diagnostic but, in combination, raise suspicion of underlying malignancy. PDAC: pancreatic ductal adenocarcinoma.

Feature	PDAC-associated new-onset diabetes	Typical type 2 diabetes	Source
Age at onset	Older, often >50 years	Any adult age; midlife common	[19,24]
Body weight	Unintentional weight loss despite hyperglycemia	Stable or increasing; overweight/obesity common	[19,20]
Glycemic course	Rapid deterioration; early insulin often needed	Gradual progression over years	[8,19]
Dominant mechanism	Beta-cell secretory failure (paraneoplastic)	Peripheral insulin resistance with compensatory hyperinsulinemia	[9,18]
Family history	Family history of pancreatic cancer raises suspicion	Family history of type 2 diabetes typical	[22]
Reversibility	May resolve after tumor resection	Not resolved by pancreatic surgery	[18]
Risk window	Highest in first 2-3 years after diabetes onset	Risk rises modestly with long-standing disease	[21,22]

Biomarkers and risk prediction: moving beyond glucose and HbA1c

Because the risk in any individual with new-onset diabetes is low, roughly 1% within three years among those over 50, current effort focuses on multivariable models and biomarkers that move beyond glucose and HbA1c [25]. Metabolic markers such as C-peptide and indices of insulin resistance reflect the inadequate beta-cell secretion characteristic of PDAC-associated diabetes but are insufficient alone for screening; however, combining unexplained weight loss with abrupt changes in HbA1c or glucose discriminates PDAC-related cases more effectively [25]. Routine laboratory values can also contribute: in a large Swedish cohort of new type 2 diabetics, low creatinine and elevated alkaline phosphatase independently predicted subsequent PDAC [26].

Tumor and inflammatory markers offer another avenue. S100A9 and galectin-3 are elevated in PDAC, disrupt insulin-driven glucose uptake, and distinguish type 3c from type 2 diabetes with area under the curves (AUCs) of 0.77 to 0.83 [27], and adding the interleukin-1 receptor antagonist to adiponectin raises discrimination to an AUC of about 0.91 [28]. CA 19-9 is the only established clinical biomarker but is inadequate alone, missing Lewis antigen-negative patients and yielding false positives in biliary obstruction [25]; multi-marker signatures generally outperform single markers, and a blood-based migration panel of tenascin-C, CA 19-9, and tissue factor pathway inhibitor separated early-stage new-onset-diabetes-related PDAC from type 2 diabetes with an AUC of 0.82 [29]. Beyond protein panels, liquid biopsy approaches, including circulating tumor DNA, DNA-methylation signatures, and extracellular vesicle-associated microRNAs, are being explored for earlier detection, but their sensitivity for early-stage, small tumors and their performance specifically within new-onset diabetes cohorts remain unproven and require prospective evaluation.

Table 2 compares the principal prediction models and biomarkers, along with their performance and validation status. Two clinical prediction models are prominent. The ENDPAC score, based on glucose change, weight change, and age at onset, had a derivation AUC of 0.87, and a threshold of 3 or more gave roughly 80% sensitivity and specificity, enriching PDAC prevalence about 4.4-fold [30]; however, external validation in 70,050 UK patients yielded an AUC of 0.73 and a positive predictive value of 0.6%, indicating that ENDPAC is better suited to triage than to diagnosis [31]. The Boursi model, incorporating laboratory, behavioral, and anthropometric data, achieved an AUC of 0.82 [32], and adding a polygenic risk score to non-genetic factors raised the C-index from 0.75 to 0.82, illustrating the value of combining data types [33]. Machine-learning approaches handling missing electronic health record data across pre-existing and new-onset diabetes achieved AUROCs of 0.78 to 0.84, outperforming ENDPAC in matched cohorts but requiring external validation and limited by very low positive predictive value [34].

**Table 2:** Risk-prediction models and biomarkers for pancreatic cancer in new-onset diabetes No tool has been shown to detect pancreatic cancer earlier or to improve survival in clinical practice. AUC: area under the curve; AUROC: area under the receiver operating characteristic curve; CA 19-9: carbohydrate antigen 19-9; EHR: electronic health record; ENDPAC: Enriching New-Onset Diabetes for Pancreatic Cancer; IL-1Ra: interleukin-1 receptor antagonist; PPV: positive predictive value; TFPI: tissue factor pathway inhibitor.

Tool	Basis/inputs	Performance (AUC; PPV)	Validation status	Key limitation	Source
ENDPAC score	Glucose change, weight change, age at onset	Derivation AUC ~0.87; external AUC ~0.73, PPV <1%	Externally validated	Poor external discrimination; triage, not diagnosis	[30,31]
Boursi model	Laboratory, behavioral, anthropometric data	AUC ~0.82	Single-study	Not prospectively validated for imaging triage	[32]
Clinical + polygenic risk score	Non-genetic factors plus polygenic score	C-index 0.75 to 0.82 with genetics added	Development	Requires external validation across populations	[33]
Machine-learning EHR models	Routine EHR variables, missing data handling	AUROC ~0.78 to 0.84	Internal	Very low PPV; needs external validation	[34]
Galectin-3/S100A9	Tumor-derived diabetogenic factors	AUC 0.77 to 0.83	Retrospective	Not prospectively validated	[27]
Adiponectin/IL-1Ra	Metabolic-inflammatory ratio	AUC ~0.91	Retrospective	Not prospectively validated	[28]
Migration panel (tenascin-C, CA 19-9, TFPI)	Multi-marker blood signature	AUC ~0.82	Retrospective	CA 19-9 misses Lewis-negative; needs validation	[29]

From primary care to early detection: can we identify high-risk patients?

New-onset diabetes warrants further evaluation, particularly when its presentation is atypical for type 2 diabetes, and raises suspicion of PDAC; because roughly 1% of people over 50 with new-onset diabetes are diagnosed with pancreatic cancer within three years, the priority is to identify higher-risk patients rather than to image everyone [19,35]. Older age at onset, unexplained weight loss, pancreatitis, a family history of pancreatic cancer, and rapidly worsening glycemia requiring early insulin are associated with increased risk, and new-onset hyperglycemia with unexplained weight loss deserves special attention because weight typically remains stable or rises in type 2 diabetes [19,21]. The ENDPAC score uses age at onset, weight change, and glucose change over the preceding year, and a score of 3 or higher may indicate increased risk, though sensitivity varies across studies; it stratifies risk rather than diagnosing PDAC and requires further prospective study before routine use [35,36].

There is currently no accepted screening strategy for sporadic PDAC based on new-onset diabetes alone [4,37]. When symptoms and risk factors warrant evaluation, multiphasic pancreas protocol CT is usually the most useful initial test, detecting a mass, ductal dilation, vascular involvement, and liver lesions and helping determine stage and resectability [37,38]. If CT is inconclusive but suspicion remains high, magnetic resonance imaging with magnetic resonance cholangiopancreatography or endoscopic ultrasound provides further evaluation; the latter also enables tissue sampling, while CA 19-9 may help when concerning imaging findings are already present but should not be used alone for screening [38,39]. Care should be coordinated across primary care, radiology, gastroenterology, and hepatopancreatobiliary specialists, with suspicious findings prompting specialist review, staging, and possible tissue sampling [37,38].

A risk-based approach may help select patients with new-onset diabetes for pancreatic testing rather than imaging all of them, which matters because PDAC is often found late, when surgery is no longer possible [36,37]. Limitations remain: new-onset diabetes may develop close to diagnosis, leaving little lead time; small or isoattenuating tumors may be missed; and some patients lack the expected weight or glucose changes, with ENDPAC scores of 3 or more showing about 56% sensitivity and 82% specificity, so the score can flag higher-risk patients but should not be used alone [35]. Imaging also carries potential harms: in a prospective pilot study of patients with new-onset diabetes, more than half had incidental extrapancreatic findings on pancreas protocol CT, which can generate anxiety, further testing, and additional resource use [40]. False positives may lead to unnecessary investigation, while a negative result should not lead clinicians to ignore new warning signs, which should prompt re-evaluation.

Looking ahead, prospective trials and richer prediction tools may clarify whether this risk-based approach improves outcomes: the Early Detection Initiative, an algorithm-based randomized trial in new-onset hyperglycemia and diabetes, is designed to test exactly this question [41], while scoring models built from routinely stored data and machine learning-optimized metabolic biomarker panels illustrate how a high-risk subgroup might be prioritized efficiently [42,43].

## Conclusions

New-onset diabetes may be an early indicator of pancreatic cancer risk, but on current evidence, it does not by itself justify universal screening. A prospective cohort confirmed an increased three-year risk of pancreatic cancer after glycemically defined new-onset diabetes that varied markedly by race and ethnicity, yet found insufficient evidence that early evaluation detects cancer earlier or improves prognosis. Imaging all patients with new-onset diabetes is feasible but problematic: a pilot study found incidental non-pancreatic findings in roughly half of patients and did not demonstrate benefit from imaging at the onset of hyperglycemia, with the single cancer detected already at an advanced stage in a small sample. Randomized, risk-stratified screening studies are therefore needed to weigh the number of patients potentially diagnosed earlier against harms such as overdiagnosis and incidental findings. Additional tools, including biomarkers, genetic information, machine learning, and readily available routine data, could help prioritize a high-risk subgroup and reduce the number of patients requiring further testing, but each still faces limitations of recalibration, modest positive predictive value, and the need for external validation across populations. In conclusion, new-onset diabetes is best regarded as a signal that, combined with biomarkers, genetic analysis, or artificial-intelligence-based models, may identify a high-risk subgroup warranting further investigation, provided these tools are validated before adoption into routine clinical practice.
